# Genome-Wide Identification and Analysis of *Brassica napus* Nonexpressor of Pathogenesis-Related Genes Family and Functional Analysis of *BnaNPR3.A02* in Clubroot Resistance

**DOI:** 10.3390/plants15203149

**Published:** 2026-10-15

**Authors:** Lin Zheng, Feixue Zhao, Ye Li, Ke Xu, Zhiting Guo, Junwei Nie, Yongji Ma, Ziyuan Chen, Hongyu Sha, Ruiheng Li, Keqi Li, Weinan Sun, Zhen Huang

**Affiliations:** State Key Laboratory of Crop Stress Biology for Arid Areas, College of Agronomy, Northwest A&F University, Yangling 712100, China

**Keywords:** *NPR3*, *Plasmodiophora brassicae*, plant immunity, allopolyploid evolution

## Abstract

Nonexpressor of pathogenesis-related (NPR) proteins are key regulators of salicylic acid (SA)-mediated plant immunity, functioning as transcriptional co-regulators. In this study, we aimed to comprehensively identify NPR family members in *Brassica napus* and its diploid progenitors, investigate their evolutionary dynamics and expression profiles, and functionally characterize the role of *BnaNPR3.A02* in clubroot resistance. We identified 23, 12, and 12 *NPR* genes in *B. napus*, *B. rapa*, and *B. oleracea*, respectively, which were phylogenetically divided into six conserved subfamilies (I–VI). Structural characterization showed that these genes possess conserved ankyrin repeats, BTB-POZ domains or the DUF3420 domains, and exhibit diversified exon–intron structures. Spatiotemporal expression profiling uncovered tissue-specific expression patterns of *NPRs* under normal conditions. Promoter cis-element analysis and transcriptomic profiling in response to phytohormones and abiotic stresses suggested broad involvement of *BnaNPRs* in environmental adaptation. GO annotation revealed that NPR1–4 members are enriched in jasmonic acid/salicylic acid-mediated defense pathways, whereas NPR5/6 members are associated with floral development. qRT-PCR analysis of 11 expressed *BnaNPR1–4* genes was performed in resistant (R) and susceptible (S) rapeseed plants upon *P. brassicae* infection. *BnaNPR3.A02* showed the greatest expression divergence, with significant induction in R plants but consistent suppression in S plants. Because NPR3 is a negative feedback regulator of SA signaling, the induction of *BnaNPR3.A02* in R plants likely reflects feedback attenuation after immune activation, whereas its suppression in S plants may indicate a defective SA signaling node. Heterologous overexpression of *BnaNPR3.A02* in Arabidopsis significantly increased disease severity and pathogen biomass, whereas Arabidopsis *npr3* knockout mutants showed enhanced resistance, supporting a conserved negative regulatory role of *NPR3* in this heterologous system. Protein–protein interaction and co-expression analyses further revealed that BnaNPR3.A02 is embedded in a stress- and defense-related network. These findings provide a foundation for understanding the evolutionary and functional divergence of the NPR family and identify *BnaNPR3.A02* as a candidate target for future functional validation and breeding of clubroot-resistant *B. napus* varieties.

## 1. Introduction

Plants must cope with various adverse environmental conditions or stresses, such as high temperature, low light, drought, and salinity. In addition, they are surrounded by various pathogens, including bacteria, fungi, protists, and insects, and have therefore evolved complex immune systems to defend against biotic stress. Plant immune responses are regulated by multiple phytohormones, including salicylic acid (SA), jasmonic acid (JA), and ethylene (ET), among which SA plays a crucial role in systemic resistance against biotrophic and hemibiotrophic pathogens [1,2,3]. The nonexpressor of pathogenesis-related (NPR) proteins are a unique class of transcriptional co-regulators in plants. As paralogous receptors for the plant defense hormone SA, they play indispensable roles in hormone-dependent plant immunity [4,5]. Plant NPR proteins are clustered into four major clades: angiosperm NPR1/2, angiosperm NPR3/4, non-seed plant NPR, and gymnosperm NPR. NPR proteins and the closely related BLADE-ON-PETIOLE (BOP) proteins collectively form the BTB-ankyrin superfamily. NPR1–4 family members typically contain BTB/POZ domains, ankyrin repeats, the DUF3420 domain, and nuclear localization signals, and they participate in SA-mediated disease resistance by interacting with transcription factors of the TGA family to modulate the expression of downstream pathogenesis-related (PR) genes [6].

In the model plant *Arabidopsis thaliana*, the NPR family comprises six NPR proteins: clade I (AtNPR1/2), clade II (AtNPR3/4), and clade III (NPR5/BOP2 and NPR6/BOP1) [7]. Clades I and II are primarily involved in defense, whereas clade III mainly functions in leaf and flower morphogenesis [8]. NPR1 functions as a central regulator of SA signaling and is essential for SA-induced expression of PR defense genes, mediating the activation of systemic acquired resistance (SAR) [9]. *NPR2* is the only paralog that can partially complement the *npr1* mutant [10]. In antagonistic regulation, its paralogs NPR3 and NPR4 act as transcriptional co-repressors that attenuate immune responses by promoting NPR1 degradation, thereby preventing resource wastage caused by excessive immune activation and maintaining immune homeostasis in plants [11,12,13]. Studies have shown that SA directly binds NPR3 and NPR4, thereby controlling NPR1 protein stability, revealing a fine-tuned regulatory relationship between NPR1 and NPR3/NPR4 [14,15].

Beyond Arabidopsis, the NPR family has been identified and functionally characterized in various crops. In wheat, 17 *NPR* genes were genome-wide identified using bioinformatics approaches. These genes exhibit a conserved evolutionary distribution pattern on chromosomes. Their promoter regions contain SA-related cis-regulatory elements, and some members show induced expression under biotic stress [16]. Targeted degradation of TaNPR3 in wheat can break immune balance and confer broad-spectrum disease resistance, providing a new strategy for disease resistance breeding [17]. In rice, alternative splicing of *OsNPR3* is promoted by the splicing regulator OsRBP11, which is targeted by bacterial TAL effectors. The resulting truncated OsNPR3 protein variant compromises defense gene activation by sequestering OsNPR1, thereby enhancing rice susceptibility to pathogens; natural variation in *OsRBP11* can restore OsNPR1 defense function and enhance rice resistance to different bacterial strains [18]. Phylogenetic analysis revealed that NPR1-like proteins are highly conserved across diverse plant species, including both monocots and dicots. In maize, the transcript levels of these genes are significantly altered in response to various abiotic stresses (cold, heat, salt, and drought), as well as pathogen infection. Furthermore, overexpression of *ZmNPR1* has been shown to enhance maize resistance against the smut fungus *Ustilago maydis* [19]. In peanut, eight *AhNPR* genes were identified and characterized. Promoter and expression analyses indicated their important roles in growth, hormone signaling, and stress responses. Overexpression of *AhNPR3A* and *AhNPR4A* significantly enhanced cold tolerance in Arabidopsis, whereas VIGS-mediated silencing of these genes led to increased cold sensitivity [20]. Furthermore, overexpression of *AtNPR1* in citrus enhanced tolerance to Huanglongbing (HLB) with a balanced immune response [21]. Expression of *AtNPR1* in chickpea activated SAR and enhanced resistance to Fusarium wilt [22]. Wang et al. conducted genome-wide identification of *NPR1*-like genes in *B. napus* and functionally characterized *BnaNPR1*. They identified 19 *BnaNPR1*-like genes and showed that knockdown of *BnaNPR1* compromised plant resistance to *Sclerotinia sclerotiorum* [23]. However, their study focused primarily on the NPR1-like subfamily and did not systematically investigate the evolutionary relationships of the full NPR family in *B. napus* and its diploid progenitors, nor did it characterize the functions of NPR3/4 members in clubroot resistance caused by *Plasmodiophora brassicae*.

*NPR* genes not only play central roles in biotic stress responses but also exhibit important functions in various abiotic stress responses. Studies have shown that constitutive expression of *AtNPR1* in transgenic rice enhances resistance to fungal and bacterial pathogens but simultaneously increases sensitivity to abiotic stresses, reflecting a trade-off between immune activation and stress tolerance [24]. In Arabidopsis, the translocation of NPR1 from chloroplasts to the nucleus activates tolerance to salt stress, and chloroplast-targeted *NPR1* overexpression enhances stress tolerance and photosynthetic capacity [25]. These findings reveal the cross-regulatory functions of *NPR* genes in biotic and abiotic stress responses, positioning them as key hubs integrating multiple stress signals.

*Brassica napus* is an important oil crop widely cultivated worldwide. However, during its growth, it faces multiple environmental and biotic challenges. Among these, clubroot disease caused by the protist *Plasmodiophora brassicae* seriously compromises the growth, development, and yield of rapeseed: infected fields typically suffer yield losses of 20–30%, and in severe cases losses can exceed 80% or result in total crop failure, causing enormous economic losses [26,27]. The disease has now spread to more than 80 countries worldwide [28]. Traditional disease control measures are ineffective; breeding and planting resistant varieties is the most effective approach. Mining disease resistance-related genes can facilitate the identification of candidate genes for breeding disease-resistant varieties.

In this study, we performed a genome-wide identification and analysis of the *NPR* gene family in *B. napus* and its diploid progenitors *B. rapa* (AA) and *B. oleracea* (CC). We systematically analyzed their phylogenetic relationships, gene structures, conserved motifs and domains, promoter cis-elements, duplication events, and expression patterns across different tissues, hormone treatments, abiotic stresses, and *P. brassicae* infection. Furthermore, we heterologously overexpressed *BnaNPR3.A02* in Arabidopsis and characterized the phenotype of the Arabidopsis *npr3* mutant, combining a heterologous gain-of-function assay with loss-of-function evidence from its Arabidopsis homolog to explore the conserved function of *NPR3* in clubroot resistance. These findings reveal the evolutionary conservation and functional divergence of NPR family members in the allopolyploid *B. napus* and its diploid progenitors *B. rapa* and *B. oleracea*, and provide evidence for a conserved negative regulatory role of *BnaNPR3.A02* in a heterologous Arabidopsis system. The results highlight *BnaNPR3.A02* as a promising candidate gene for future mechanistic investigation and crop improvement in rapeseed.

## 2. Results

### 2.1. Identification and Characterization of NPR Family Genes in B. napus, B. rapa, and B. oleracea

To elucidate the evolutionary history of the NPR family, we performed a systematic genome-wide identification and analysis. Using *AtNPR1–6* homologs and the Pfam conserved domain model (PF00651) as queries, BLASTP and HMMER searches were conducted against the proteomes of *B. napus*, *B. rapa*, and *B. oleracea*. After stringent filtering, 23, 12, and 12 *NPR* genes were identified in *B. napus*, *B. rapa*, and *B. oleracea,* respectively (Supplementary Table S1). These genes were named based on their sequence similarity to Arabidopsis *NPR* homologs. Although *B. napus* (AACC) originated from the hybridization and polyploidization of its diploid progenitors *B. rapa* (AA) and *B. oleracea* (CC), the total number of NPR family members in *B. napus* was slightly lower than the sum of those in the two diploid parents. This slight discrepancy suggests that the NPR family experienced limited gene loss following polyploidization, likely because these genes are functionally essential and subject to dosage balance constraints.

We analyzed the physicochemical properties of NPR proteins from Arabidopsis and the three Brassica species. The results showed that amino acid lengths ranged from 291 to 1022 aa (average 532 aa), molecular weights from 32.58 to 114.71 kDa (average 59.44 kDa), and isoelectric points (pI) from 4.59 to 8.07 (average 6.02). Notably, the instability index of all 53 NPR proteins was greater than 40, indicating that these NPR proteins are predicted to be unstable. Subcellular localization prediction revealed diverse intracellular distributions: 13 proteins were localized in the nucleus, 20 in chloroplasts, 18 in the cytoplasm, one (BnaNPR1.C08) in both the cytoplasm and nucleus, and one (BolNPR1.C01) in the plasma membrane (Supplementary Table S1). This diversity suggests that NPR family proteins may participate in multiple cellular functions.

### 2.2. Phylogenetic Analysis of NPR Family Genes in Three Brassica Species

**Figure 1:**
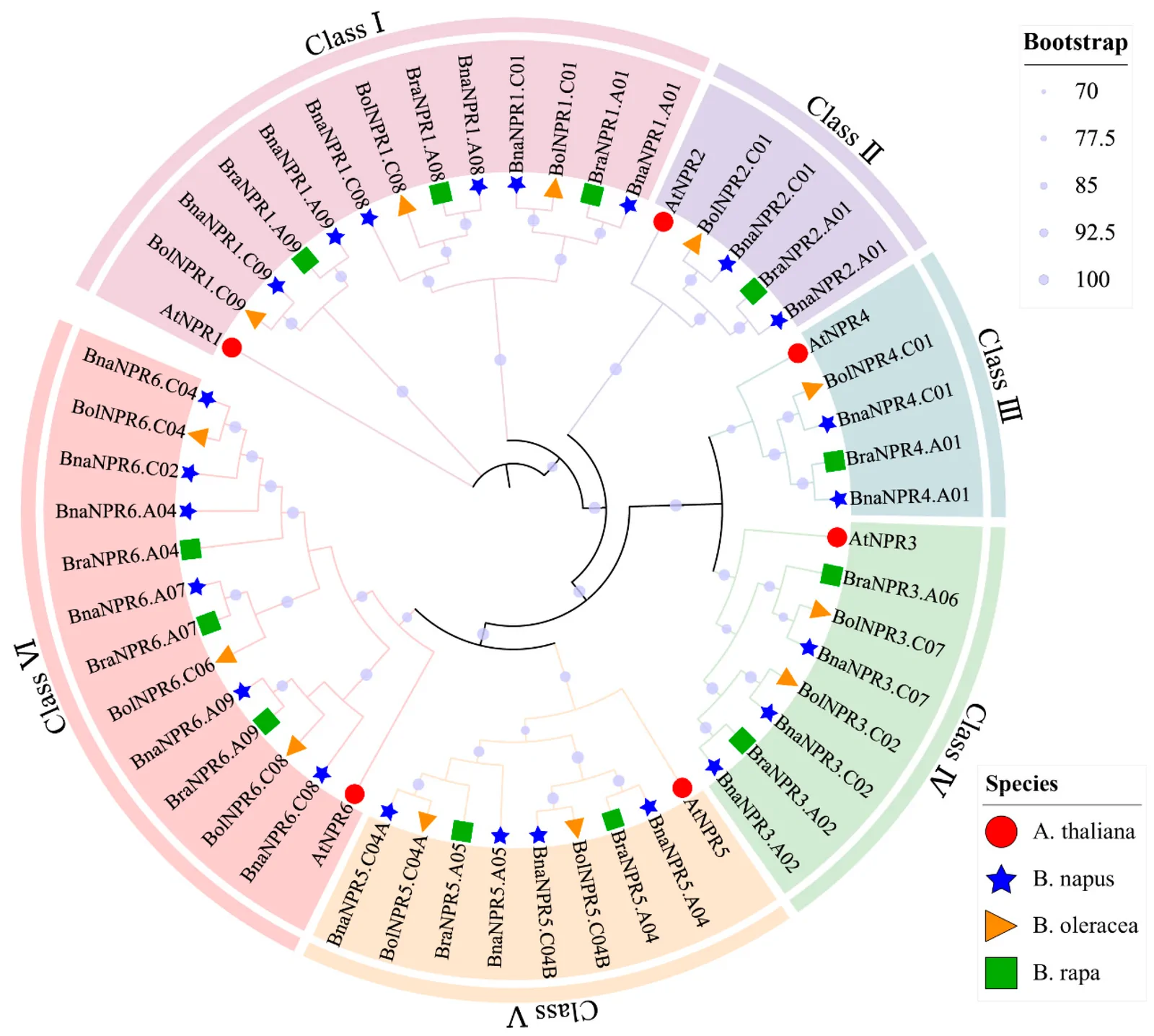
Phylogenetic relationships of NPR proteins from *A. thaliana*, *B. napus*, *B. rapa*, and *B. oleracea*. The maximum likelihood (ML) tree was constructed using the amino acid sequences of 53 NPR proteins. The six subfamilies (I–VI) are highlighted in different colors. Bootstrap values (≥70%) from 1000 replicates are shown at the nodes.

To explore the evolutionary relationships of *NPR* genes in Arabidopsis, *B. napus*, *B. rapa*, and *B. oleracea*, a maximum likelihood (ML) phylogenetic tree was constructed using 53 NPR proteins from these four species (Figure 1). The results showed that *NPR* genes were divided into six subfamilies (Subfamilies I–VI), consistent with the established classification in Arabidopsis. The six subfamilies comprised 13, 5, 5, 8, 9, and 13 members, respectively. Notably, all six subfamilies included genes from all four species, with no species-specific clades. Moreover, the homologs in *B. napus* clustered with those from its diploid progenitors—*B. rapa* (the A-genome donor) and *B. oleracea* (the C-genome donor)—further supporting the hybrid origin and allopolyploid history of *B. napus*.

### 2.3. Conserved Motifs, Conserved Domains and Gene Structure Analysis of NPR Family Genes

**Figure 2:**
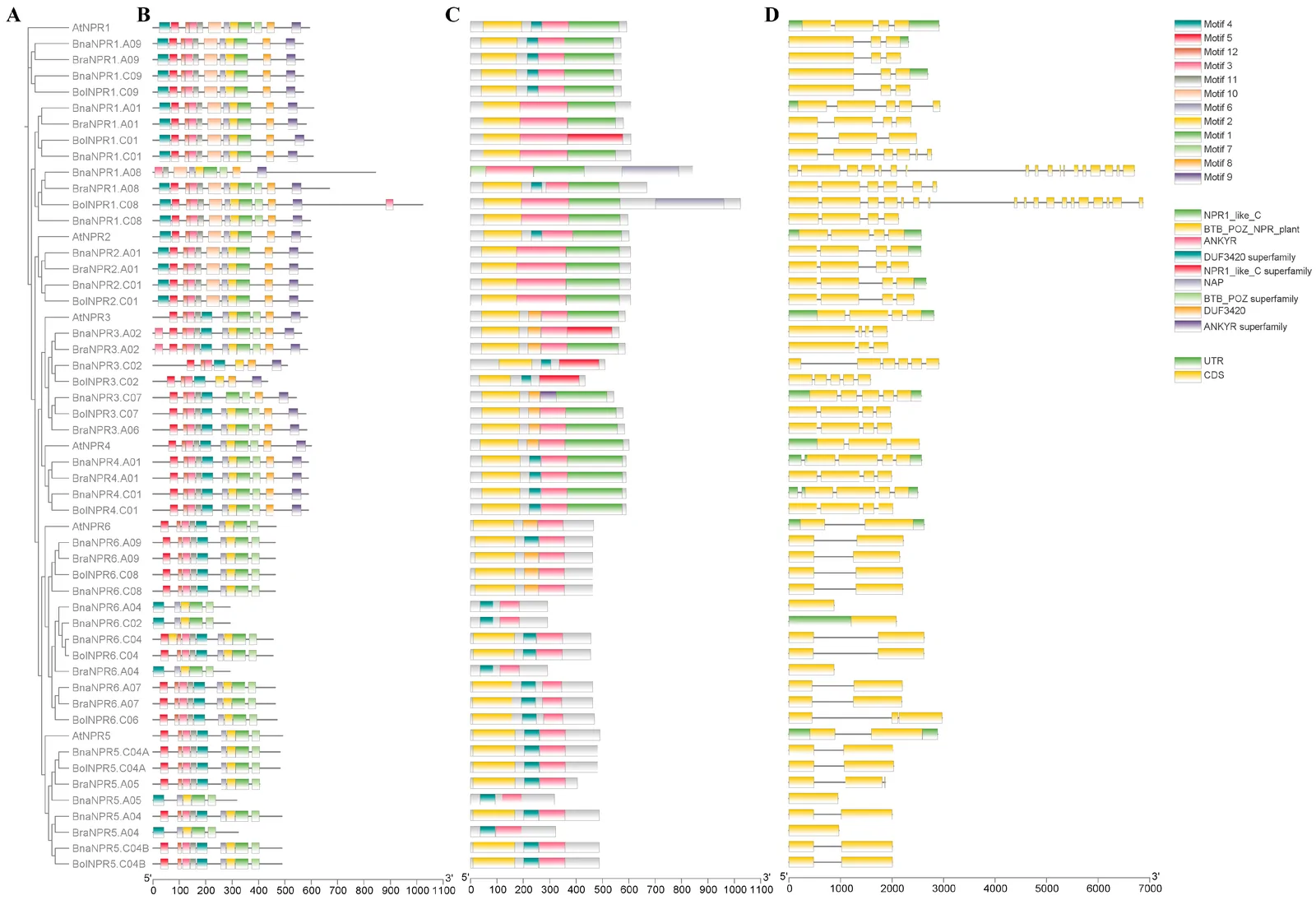
Conserved motifs, domains and gene structures of NPR genes in *A. thaliana* and three Brassica species. (**A**) Phylogenetic tree of 53 NPR genes. (**B**) Distribution of conserved motifs (1–12) identified by MEME. Different motif patterns are indicated by colored boxes. (**C**) Conserved domain architectures annotated by Batch CDD-search. (**D**) Exon–intron structures. Exons, introns and untranslated regions (UTRs) are represented by yellow boxes, black lines and green boxes, respectively. The horizontal axis indicates the gene length (bp).

To investigate the functional specialization and evolutionary diversity of NPR proteins, conserved motif analysis, domain annotation, and gene structure analysis were performed. MEME analysis identified 12 conserved motifs among the 53 NPR proteins (Supplementary Figure S1). The distribution of these motifs varied considerably among different subfamilies. Motif 4 was the most prevalent, present in 52 proteins (98.1%) and absent only in BnaNPR1.A08. Motifs 5 and 12 were detected in 47 proteins (88.7%) and were absent in six proteins: BnaNPR1.A08, BnaNPR6.A04, BnaNPR6.C02, BraNPR6.A04, BnaNPR5.A05, and BraNPR5.A04. Motif 3 was found in 48 proteins (90.6%) and was absent in five of the above six proteins (BnaNPR6.A04, BnaNPR6.C02, BraNPR6.A04, BnaNPR5.A05, and BraNPR5.A04). Motif 11 was present in 46 proteins (86.8%) and was absent in the same five proteins plus BnaNPR3.C02 and BolNPR3.C02. In contrast, Motif 10 was the most restricted and was found exclusively in members of the NPR1 and NPR2 subfamilies. Losses of Motifs 6, 1, and 2 were confined to the NPR3 subfamily: Motif 6 was absent in BnaNPR3.C02, BolNPR3.C02, and BnaNPR3.C07; Motif 1 was absent in BnaNPR3.C02 and BolNPR3.C02; and Motif 2 was absent only in BnaNPR3.C07. Motif 7 was detected in 38 proteins (71.7%) and was present in all members of the NPR4, NPR5, and NPR6 subfamilies, whereas Motifs 8 and 9 were found exclusively in NPR1–NPR4 subfamily members. With regard to positional distribution, Motifs 4 and 5 were predominantly located at the N-terminus, whereas Motifs 7, 8, and 9 were mainly found at the C-terminus. These results indicate that each subfamily exhibits a distinct but conserved motif composition, suggesting that proteins within the same subfamily may share similar functions (Figure 2B).

Domain composition analysis revealed that BnaNPR6.A04, BnaNPR6.C02, BraNPR6.A04, BnaNPR5.A05, and BraNPR5.A04 contained the DUF3420 domain, whereas the remaining 48 NPR proteins carried a BTB/POZ domain. All members of Subfamilies I–IV possessed the NPR1_like_C domain or belonged to the NPR1_like_C superfamily. Except for BnaNPR3.C02 and BolNPR3.C02, all other NPR proteins contained ankyrin repeats. Differences in motif distribution and domain composition among subfamilies further supported the functional diversification of the NPR family (Figure 2C).

Gene structure analysis revealed that the number of exons in *NPR* genes varied considerably, ranging from 1 to 18. The most prevalent gene structures contained either 2 or 4 exons, representing 32 genes in total. *BnaNPR1.A08* and *BolNPR1.C08* possessed the highest number of exons, each containing 18 exons. Notably, all members of the NPR5 and NPR6 subfamilies possessed only one or two exons, indicative of relatively simple gene structures within these two subfamilies. Overall, *NPR* genes exhibited substantial structural diversity, which was consistent with the variation in motif and domain composition described above, further supporting potential functional divergence among subfamilies (Figure 2D).

### 2.4. NPR Family Genes’ Distribution and Syntenic Analyses

**Figure 3:**
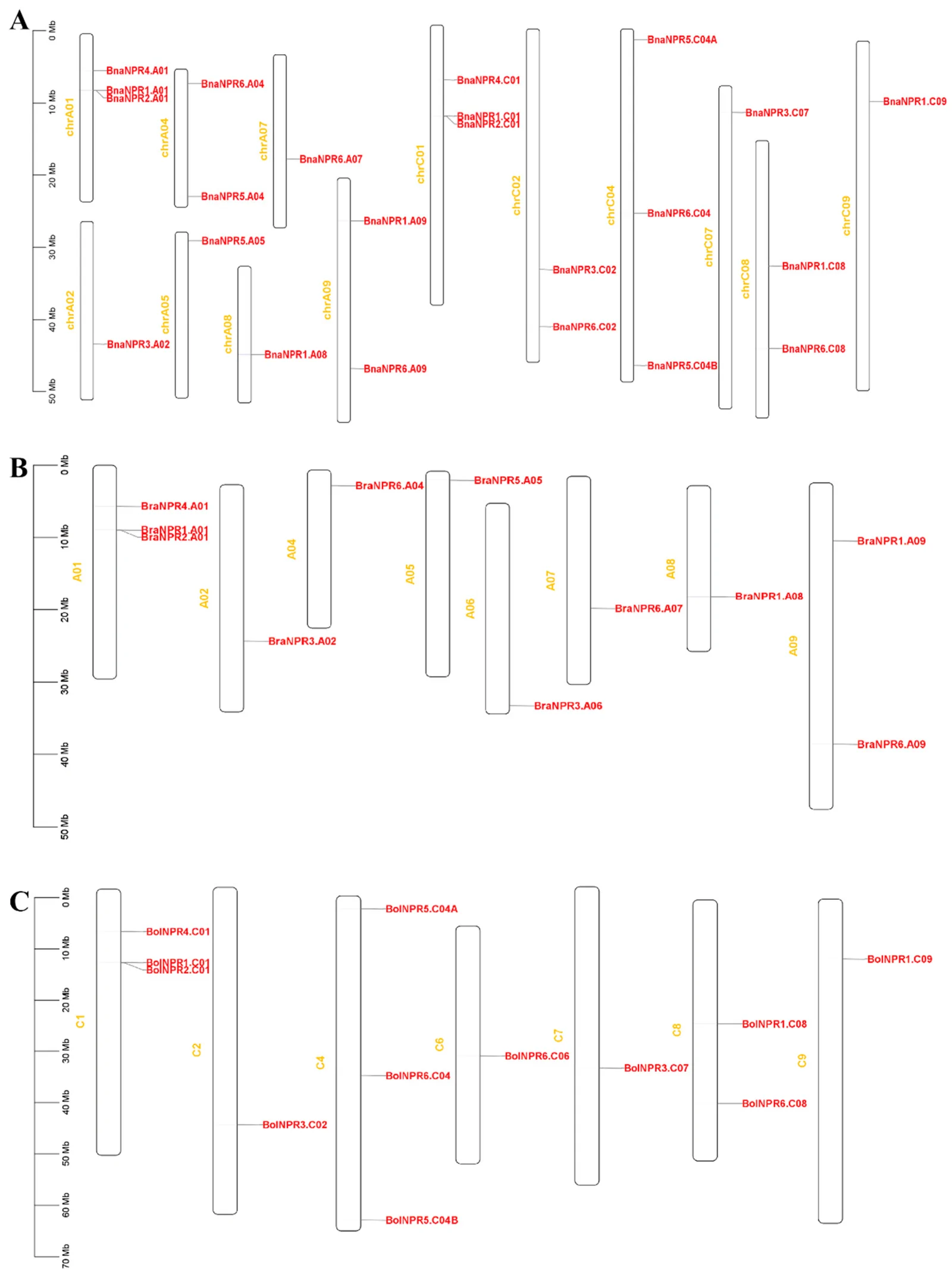
Chromosomal distribution of *NPR* genes in *Brassica napus* (**A**), *Brassica rapa* (**B**), and *Brassica oleracea* (**C**). Chromosomes are shown as gray bars, with chromosome numbers indicated on the left. The scale bar represents megabases (Mb). Gene names are placed to the right of each chromosome.

The chromosomal distribution of *NPR* genes in *B. napus*, *B. rapa*, and *B. oleracea* is shown in Figure 3. A total of 23 *BnaNPR* genes were distributed on 13 chromosomes, with 11 located on the A subgenome and 12 on the C subgenome. Chromosomes A01 and C01 harbored the highest number of *BnaNPR* genes (three each). Twelve *BraNPR* genes were distributed on 8 chromosomes in *B. rapa*, and 12 *BolNPR* genes on 7 chromosomes in *B. oleracea*.

**Figure 4:**
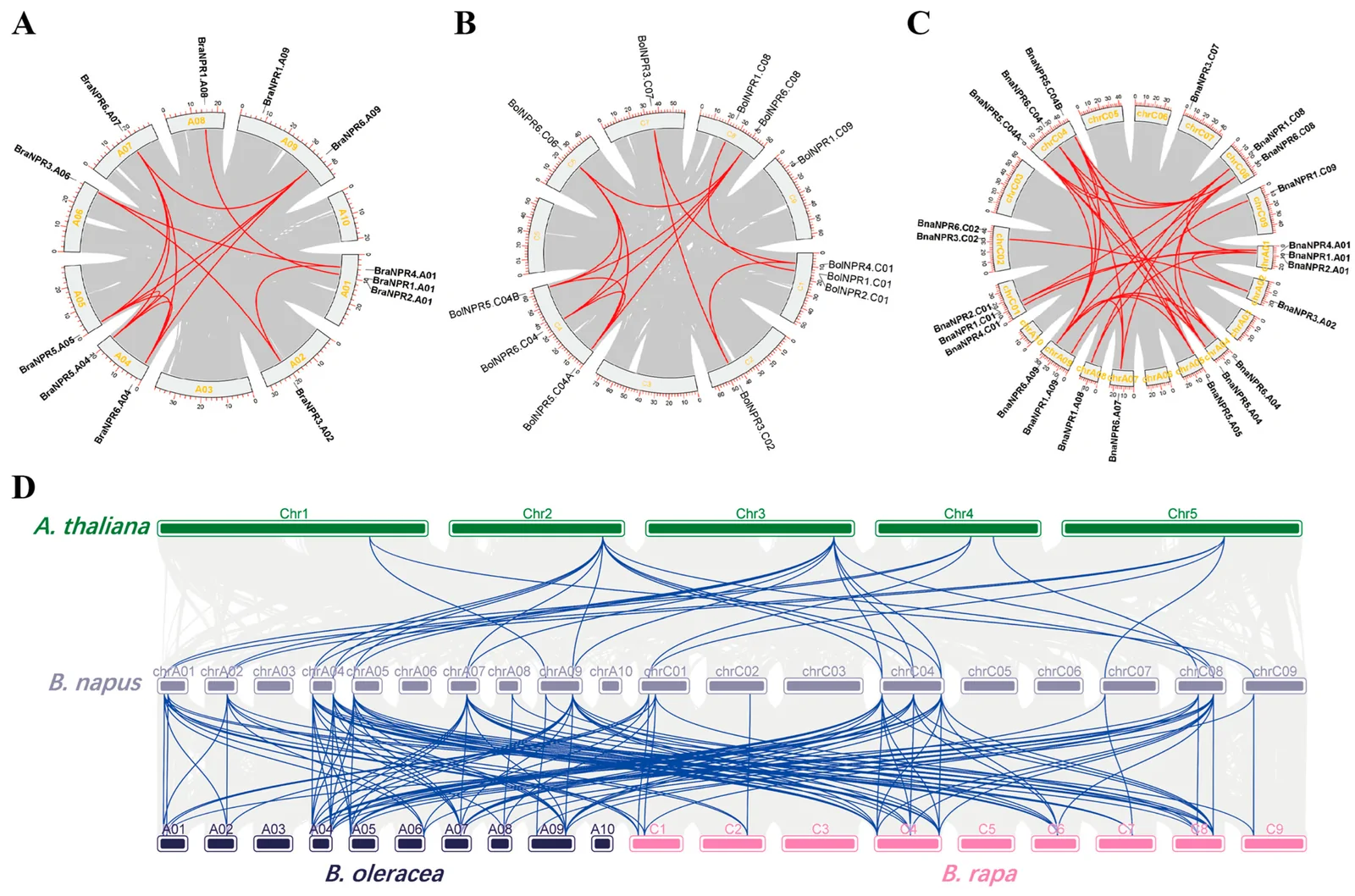
Synteny analysis of *NPR* genes among three Brassica species and *Arabidopsis thaliana*. (**A**) Intra-species synteny in *B. rapa*. (**B**) Intra-species synteny in *B. oleracea*. (**C**) Intra-species synteny in *B. napus*. Gray lines in the background represent all syntenic blocks, and red lines highlight NPR gene pairs. (**D**) Inter-species synteny among *A. thaliana*, *B. napus*, *B. rapa* and *B. oleracea*. Syntenic connections of *NPR* genes are indicated by blue lines, with gray lines representing the background syntenic blocks.

To elucidate the evolutionary processes of *NPR* genes, we analyzed gene duplication events using MCScanX. A total of 14, 13, and 31 pairs of segmentally duplicated genes were identified in *B. rapa*, *B. oleracea*, and *B. napus*, respectively (Figure 4A–C; Supplementary Table S2). Inter-species synteny analysis revealed that 16 *BnaNPR* genes were homologous to 11 *BraNPR* genes, *20 BnaNPR* genes to 11 *BolNPR* genes, and 18 *BnaNPR* genes to 6 *AtNPR* genes, generating a total of 147 syntenic gene pairs. Notably, *BnaNPR* genes located on the A subgenome of *B. napus* showed homology not only to the A genome of *B. rapa* but also to the C genome of *B. oleracea*; similarly, *BnaNPR* genes on the C subgenome also exhibited homology to both diploid progenitor genomes. These findings suggested extensive syntenic relationships between the A and C subgenomes (Figure 4D).

To evaluate the selective pressure acting on *NPR* genes from the three Brassica species, we calculated the ratio of non-synonymous (Ka) to synonymous (Ks) substitutions. The Ka/Ks ratios of all 205 gene pairs were less than 1, indicating that these genes had undergone purifying selection after segmental duplication. This selection regime was crucial for the expansion of the NPR family (Supplementary Table S2).

### 2.5. Prediction of Cis-Regulatory Elements in the Promoter of NPR Family Genes in Three Brassica Species

**Figure 5:**
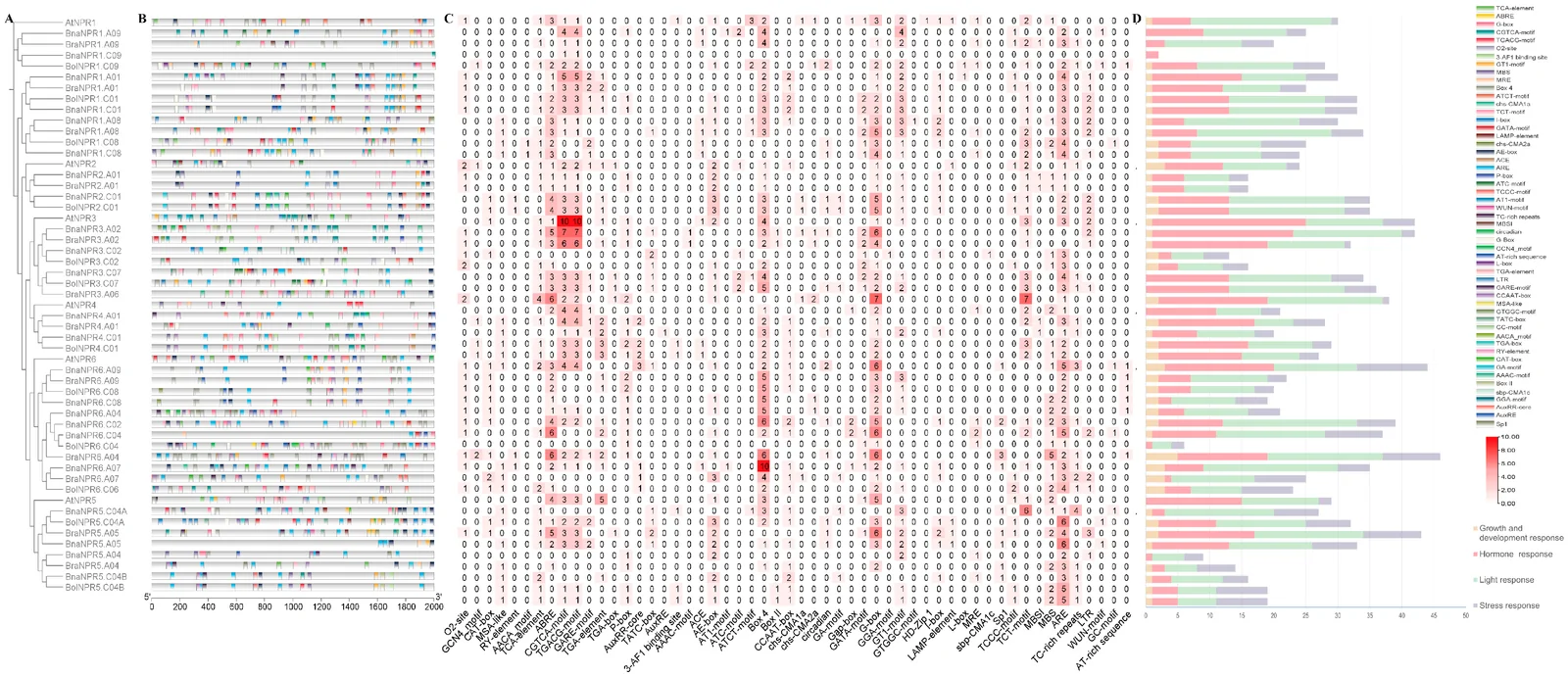
Cis-regulatory elements (CREs) in the promoters of NPR genes. (**A**) Phylogenetic tree of 53 NPR proteins. (**B**) Distribution of CREs in the 2 kb upstream regions of NPR genes; different element types are indicated by colored boxes. (**C**) Heatmap showing the number of individual CREs per gene. (**D**) Bar plot showing the total number of CREs classified into four major categories: hormone-responsive, stress-responsive, developmental, and light-responsive elements.

To investigate the transcriptional regulatory mechanisms of *NPR* genes, we analyzed the cis-acting regulatory elements (CREs) within the 2 kb upstream regions of 53 *NPR* genes. A variety of hormone-responsive, development-related, light-responsive, and stress-responsive elements were identified (Figure 5B).

Hormone-responsive elements were widely distributed in *NPR* gene promoters, including auxin-responsive elements (TGA-element, AuxRR-core, TGA-box, AuxRE), gibberellin-responsive elements (TATC-box, GARE-motif, P-box), salicylic acid-responsive elements (TCA-element), abscisic acid-responsive elements (ABRE), and methyl jasmonate-responsive elements (CGTCA-motif and TGACG-motif). Among these, ABRE, CGTCA/TGACG motifs, and TCA-element were present in the majority of *NPR* genes, suggesting that *NPR* genes may be coordinately regulated by multiple hormone signaling pathways. Development-related elements were detected in most *NPR* gene promoters, including MRE, AACA-motif, MBSI, CCAAT-box, MSA-like, RY-element, O2-site, CAT-box, GCN4-motif, and HD-Zip 1. MSA-like elements were predominantly distributed in NPR5 and NPR6 subfamily members, implying their potential involvement in cell cycle regulation. Light-responsive elements were highly enriched in *NPR* gene promoters, including G-box, Box 4, GT1-motif, Sp1, AE-box, TCT-motif, I-box, GATA-motif, ATCT-motif, and ACE, indicating that *NPR* gene expression may be modulated by light signals.

In addition, most *NPR* gene promoters harbored various stress-responsive elements, including anaerobic induction-related elements (ARE, GC-motif), defense and stress response elements (TC-rich repeats), drought-responsive elements (MBS), low-temperature-responsive elements (LTR), and wound-responsive elements (WUN-motif). The distribution and abundance of these elements varied considerably, with ARE being the most widely distributed, whereas GC-motif and WUN-motif were relatively restricted. These results suggest that *NPR* genes may play important roles in responses to multiple environmental stresses. Collectively, the diversity of CREs in *NPR* gene promoters implies that these genes are subject to sophisticated transcriptional regulation by hormonal, developmental, light, and stress signals.

### 2.6. GO Annotation of BnaNPRs

**Figure 6:**
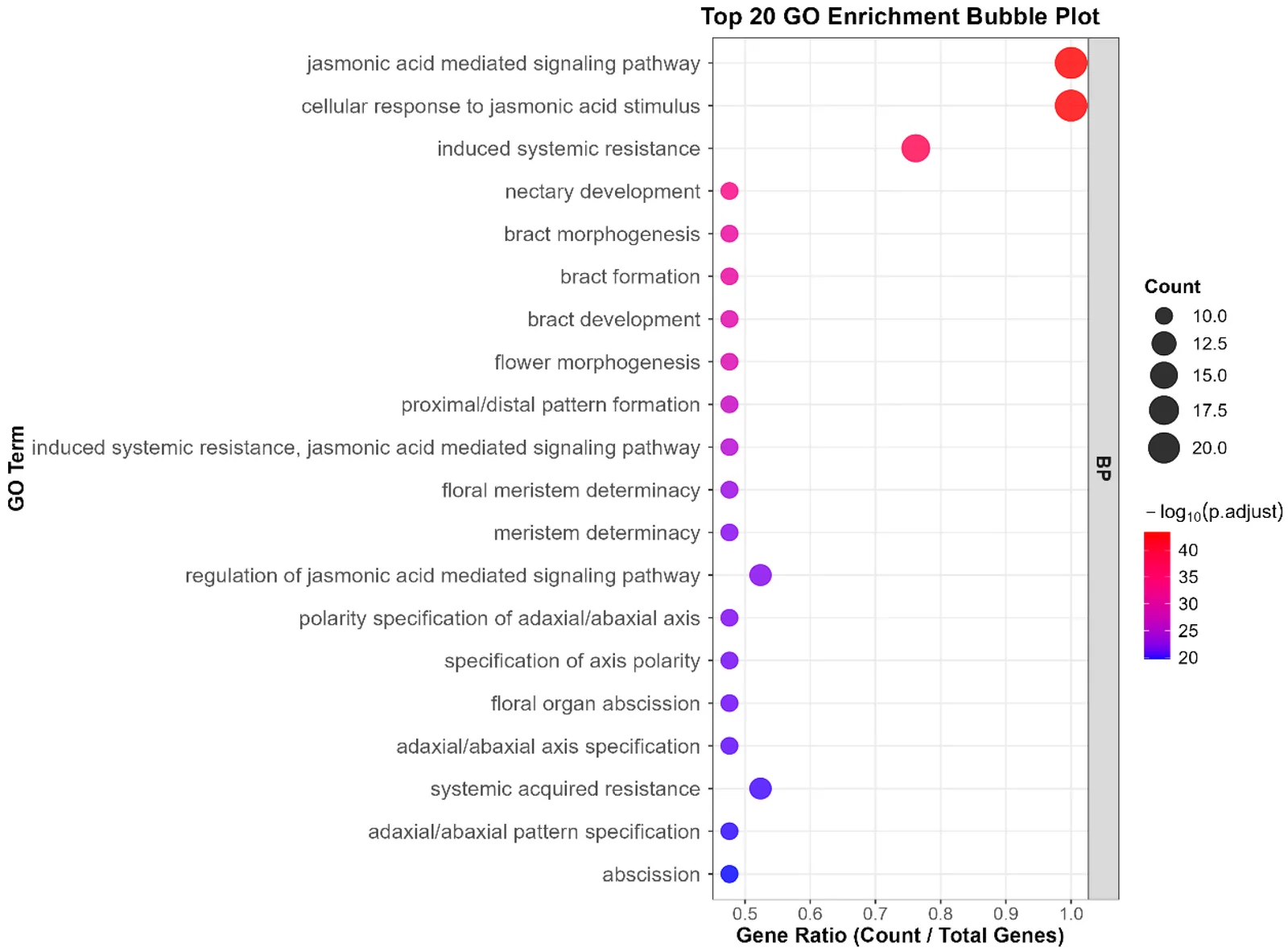
Gene Ontology (GO) enrichment analysis of *BnaNPR* genes (top 20 significantly enriched terms). The x-axis represents the gene ratio, and the y-axis lists the GO terms. Dot size is proportional to the number of annotated genes, and color intensity indicates the significance level (−log10 P-adjust).

To investigate the functions of *BnaNPR* genes, Gene Ontology (GO) annotation and enrichment analyses were performed on 21 of the 23 *BnaNPR* genes. *BnaNPR1.C09* and *BnaNPR6.C02* were excluded from the GO analysis because they lacked annotated transcript models in the BnIR genome assembly. The top 20 most significantly enriched GO terms were all assigned to the biological process (BP) category (Figure 6). In the biological process (BP) category, the most significantly enriched terms included the jasmonic acid-mediated signaling pathway, cellular response to jasmonic acid stimulus, induced systemic resistance, and systemic acquired resistance and its regulation, and regulation of JA- and SA-mediated signaling pathways. The enrichment of these defense-related GO terms corresponds to the immune functions of Arabidopsis clade I/II (AtNPR1/2/3/4). In addition, several terms associated with flower and bract development were identified, including nectary development, bract morphogenesis/formation/development, flower morphogenesis, adaxial/abaxial axis specification, and floral organ abscission, corresponding to the developmental regulatory functions of Arabidopsis clade III (AtNPR5/BOP2 and AtNPR6/BOP1). Immune-related terms such as activation of innate immune response and positive regulation of immune response were also significantly enriched. In conclusion, *BnaNPR* genes can be divided into two major functional groups: one involved in JA/SA-mediated defense signaling and immune regulation, and the other involved in floral organ development and morphogenesis. This functional divergence is highly consistent with the division of the Arabidopsis NPR family into clade I/II and clade III, suggesting that *BnaNPR* genes have largely retained the functional characteristics of their Arabidopsis orthologs during evolution.

### 2.7. Spatial and Temporal Expression Patterns of BnaNPRs

To explore the developmental expression dynamics of *BnaNPR* genes, transcriptomic data from the BnIR database were analyzed across various tissues, including flower buds (2 mm, 4 mm), filament, petal, pollen, sepal, cotyledon, vegetative rosette leaf, root, upper/middle/lower stem epidermis, leaves (leaf 1 to leaf 23 stages), seeds (14–64 days after flowering, DAF), and siliques (2–60 DAF) (Supplementary Figures S2 and S3). As noted above, *BnaNPR1.C09* and *BnaNPR6.C02* were not included in this analysis because they lacked annotated transcript models in the current BnIR genome assembly. Overall, *BnaNPR* genes exhibited distinct tissue-preferential and developmentally regulated expression patterns, reflecting functional diversification within the family.

Tissue expression analysis revealed that *BnaNPR1.C01*, *BnaNPR2.A01*, *BnaNPR2.C01*, and *BnaNPR4.A01* were predominantly expressed in roots and stem epidermis, whereas *BnaNPR3.C07* showed preferential expression in petals and sepals. Members of the *BnaNPR6* subfamily were mainly enriched in floral organs, particularly in petals and sepals, with extremely low expression in vegetative tissues. *BnaNPR5* members exhibited diverse patterns, with some preferentially expressed in roots or during late seed development. During leaf development, the transcript levels of most *BnaNPR1*, *BnaNPR2*, and *BnaNPR4* genes gradually increased. Similarly, *BnaNPR1.C01*, *BnaNPR2.C01*, *BnaNPR3.C07*, and *BnaNPR4.A01/C01* showed marked upregulation during seed and silique maturation. Overall, the dynamic and tissue-specific expression profiles of *BnaNPR* genes across various organs and developmental stages suggest their broad functional diversity throughout the entire growth and reproductive cycle of *B. napus*.

### 2.8. Expression of BnaNPRs Under Phytohormone Treatment

Phytohormones are key regulators of growth, development, and stress adaptation. We analyzed the transcriptomic responses of *BnaNPR* genes to seven phytohormones (IAA, ACC, GA, ABA, TZ, JA-Me, and BL) in leaves and roots (Supplementary Figure S4). In leaves, JA acted as a broad suppressor, attenuating the recovery of multiple BnaNPR1/2/4 members at later time points and consistently downregulating *BnaNPR4.A01* and *BnaNPR3.C07*. Conversely, ACC and BL exhibited strong inductive effects, rapidly upregulating *BnaNPR3.C07* and *BnaNPR4.A01/C01* or triggering early peaks of *BnaNPR1.C01* and *BnaNPR4.A01*. ABA displayed a time-dependent dual regulatory mode: it induced *BnaNPR1.A01/C01* at 1 h but suppressed *BnaNPR4.A01/C01* and *BnaNPR1.A08/A09* at 3 h, while also blocking the late-stage upregulation of *BnaNPR1.C01*, *BnaNPR6.C04*, and *BnaNPR2.A01* observed in the controls. TZ broadly induced members of the BnaNPR5 and BnaNPR6 subfamilies, yet concurrently suppressed *BnaNPR4.A01* and *BnaNPR1.C01*. IAA moderately induced *BnaNPR4.A01* and *BnaNPR1.A01/C01* at 3 h, whereas GA primarily suppressed the transient increase in *BnaNPR1.C01* and *BnaNPR3.C07* (Supplementary Figure S4A).

In roots, the basal expression levels of *BnaNPR4.A01*, *BnaNPR4.C01*, and *BnaNPR1.C01* were substantially higher than those in leaves, and hormone-induced changes were more pronounced. IAA and GA strongly induced *BnaNPR5.A04* and *BnaNPR5.C04B*; IAA additionally upregulated *BnaNPR3.C07*. ABA and JA exerted broad suppression in roots, reducing the transcript levels of most BnaNPR1/2/4/5 members. TZ specifically activated *BnaNPR6.A09* and *BnaNPR6.C08*. ACC primarily induced *BnaNPR2.A01* but suppressed *BnaNPR5.A04* and *BnaNPR5.C04B*. BL induced *BnaNPR3.C07* and *BnaNPR4.A01* while suppressing the 0.5-h peak of *BnaNPR1.C01*. GA induced *BnaNPR1.A01* to reach its highest level at 6 h (Supplementary Figure S4B). Taken together, *BnaNPR* genes responded to different phytohormones in a gene-specific and tissue-specific manner. Notably, JA acted as a broad suppressor, whereas ACC and BL displayed strong inductive effects, particularly in leaves. These results suggest that BnaNPRs may participate in the crosstalk of multiple hormone signaling pathways.

### 2.9. Expression Profiles of BnaNPRs Under Abiotic Stresses

We also investigated the responses of *BnaNPR* genes in various abiotic stresses, including salt (200 mM NaCl), drought (exposure to an air stream), freezing (–4 °C), cold (4 °C), heat (38 °C), and osmotic stress (300 mM mannitol), using transcriptomic data from the BnIR database (Supplementary Figure S5A,B). Under control conditions, most *BnaNPR* genes maintained relatively stable expression levels across the 0–24 h time course. BnaNPR1/2/4 members were the most responsive, whereas BnaNPR5/6 members showed relatively weak or negligible changes under most stresses.

Drought stress triggered the strongest and most extensive response, with 8 out of 21 *BnaNPR* genes significantly upregulated in leaves, including *BnaNPR1.A01/C01*, *BnaNPR2.A01/C01*, *BnaNPR3.A02/C07*, and *BnaNPR4.A01/C01*, among which *BnaNPR4.A01* showed the most pronounced induction. The root response to drought was much weaker, with only *BnaNPR3.C07* upregulated. Salt stress induced a distinctly different pattern: *BnaNPR1.A01/C01*, *BnaNPR2.A01/C01*, and *BnaNPR3.A02* were upregulated, whereas *BnaNPR3.C07*, *BnaNPR4.A01*, and *BnaNPR4.C01* were repressed in leaves. Roots showed activation of *BnaNPR2.A01/C01* and *BnaNPR3.C07*, alongside marked downregulation of BnaNPR5/6 members. Under osmotic stress, *BnaNPR2.A01/C01* were specifically induced in leaves, and *BnaNPR1.C01* and *BnaNPR4.A01/C01* showed transient early induction but were suppressed at later stages. *BnaNPR3.C07* was the only gene induced in roots.

Temperature extremes provoked highly specific expression patterns. Under freezing stress (−4 °C), leaves showed upregulation of *BnaNPR1.A08*, *BnaNPR1.C08*, and *BnaNPR2.A01/C01*, while five members—*BnaNPR1.A01*, *BnaNPR1.C01*, *BnaNPR3.C07*, *BnaNPR4.A01*, and *BnaNPR4.C01*—were repressed. In roots, only *BnaNPR5.A04* was induced. Under cold stress (4 °C), a distinct pattern was observed: in leaves, *BnaNPR1.A08*, *BnaNPR1.C08*, and *BnaNPR2.A01/C01* showed transient early induction, accompanied by downregulation of five other members; in roots, *BnaNPR2.A01/C01*, *BnaNPR5.A04*, and *BnaNPR5.C04B* were upregulated, whereas *BnaNPR1.A01/C01*, *BnaNPR3.C07*, and *BnaNPR4.A01/C01* were repressed. Heat stress had the weakest effect, inducing *BnaNPR1.A01/C01* and repressing *BnaNPR4.A01/C01* and *BnaNPR3.C07* in leaves, with no significant changes in roots. These results suggest that *BnaNPR* genes may play important roles in mediating plant responses to various abiotic stresses and contribute to the stress tolerance mechanisms of *B. napus*.

### 2.10. Expression Profiles of BnaNPRs Under P. brassicae Stress

*Plasmodiophora brassicae* is a devastating pathogen that causes clubroot disease in rapeseed (*Brassica napus*). Given the significant enrichment of NPR1–4 members in immunity-related GO terms and their differential expression responses to multiple phytohormones and abiotic stresses, this study further focused on BnaNPR1–4 to investigate their expression dynamics in response to *P. brassicae* infection. qRT-PCR was performed on the rapeseed line “1603” at various time points post-inoculation to explore the potential regulatory roles of these genes in clubroot resistance. Among the 13 *BnaNPR1-4* genes, two members (*BnaNPR3.C02* and *BnaNPR1.C09*) exhibited consistently high Ct values across all samples, indicating extremely low or undetectable transcript abundance, and were therefore excluded from further analysis. The remaining 11 genes showed no significant changes in the non-inoculated control across all time points, whereas distinct expression patterns emerged between the R and S plants upon infection.

**Figure 7:**
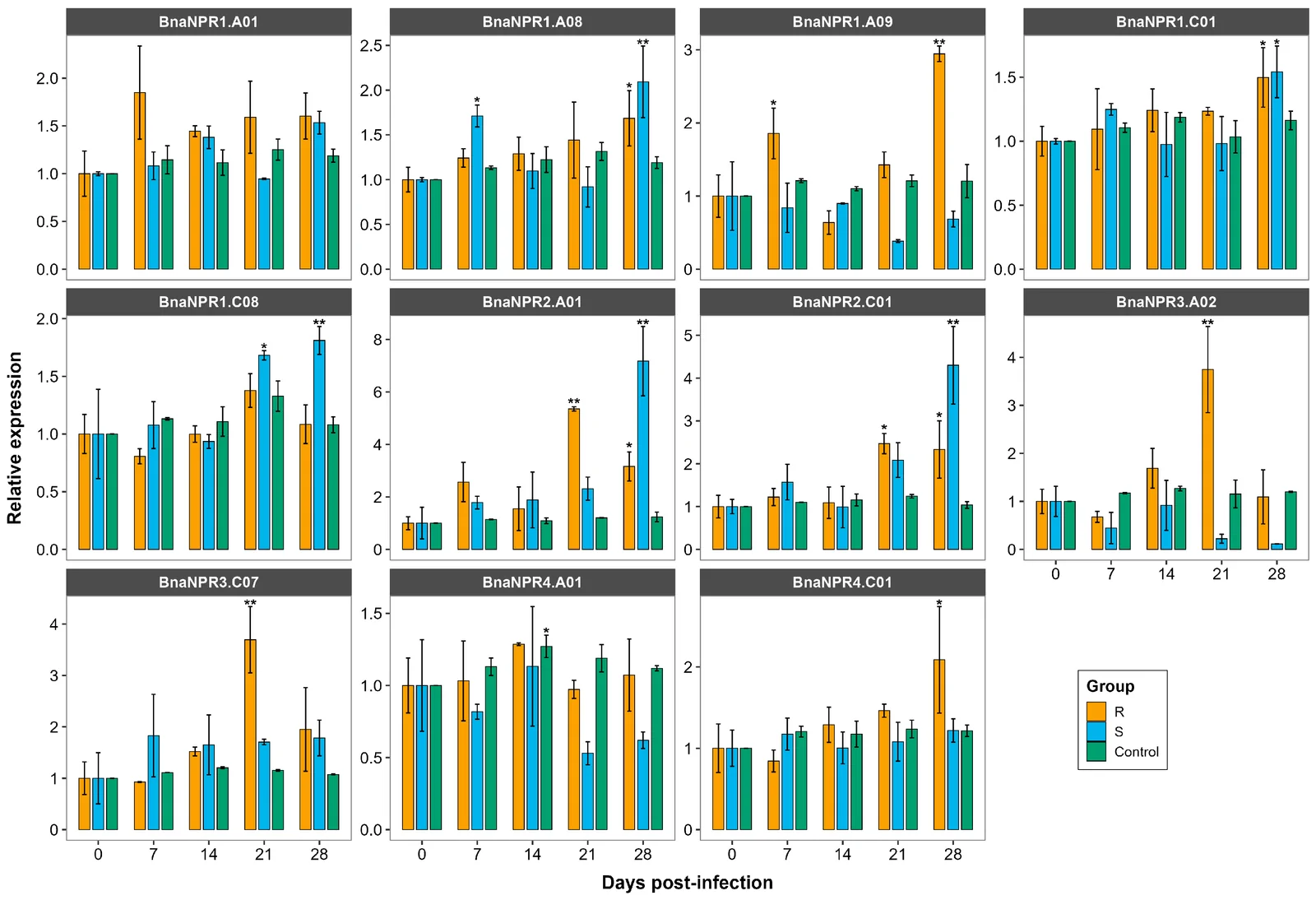
Expression profiles of *BnaNPR* genes in response to *Plasmodiophora brassicae* infection. Relative expression levels of 11 *BnaNPR* genes in resistant (R) and susceptible (S) rapeseed line “1603” at 0, 7, 14, 21, and 28 days post-inoculation (dpi). Values are means ± SD from three biological replicates. For each genotype, one-way ANOVA with Tukey’s HSD post-hoc test was performed for multiple comparisons across time points; asterisks indicate significant differences between the indicated time point and the 0 dpi control within the same genotype (* p < 0.05, ** *p* < 0.01).

The five NPR1 members (*BnaNPR1.A01*, *A08*, *A09*, *C01*, and *C08*) displayed broadly similar expression trends between R and S plants. *BnaNPR1.A01* showed no significant changes in either R or S plants. *BnaNPR1.A08* exhibited a sustained upward trend in R plants, whereas in S plants it was extremely significantly upregulated at both 7 and 28 dpi. *BnaNPR1.A09* followed a V-shaped pattern in R plants, with extreme upregulation at 7 and 28 dpi, but showed slight suppression in S plants. *BnaNPR1.C01* was significantly upregulated at 28 dpi in both R and S plants. *BnaNPR1.C08* remained stable across all time points in R plants, whereas in S plants it displayed a progressive increase, reaching significance at 21 dpi and extreme significance at 28 dpi. Both *BnaNPR2.A01* and *BnaNPR2.C01* exhibited a rise-and-fall pattern in R plants, peaking at 21 dpi and remaining significantly elevated at 28 dpi relative to 0 dpi. In contrast, they showed a sustained upward trajectory in S plants, reaching extremely significant differences at 28 dpi. NPR3 members displayed strict resistance-specific induction. *BnaNPR3.A02* and *BnaNPR3.C07* were continuously upregulated from 7 to 21 dpi in R plants. In S plants, *BnaNPR3.A02* was consistently repressed throughout the infection course, whereas *BnaNPR3.C07* maintained stable expression levels (1.65- to 1.83-fold relative to 0 dpi) with no significant differences at any time point. *BnaNPR4.A01* showed no significant changes in R plants, but was downregulated at later stages in S plants. *BnaNPR4.C01* exhibited resistance-specific upregulation, with a sustained increasing trend in R plants, while remaining stable and non-significant throughout the infection course in S plants (Figure 7).

Expression pattern analysis revealed that three genes—*BnaNPR3.A02*, *BnaNPR3.C07*, and *BnaNPR4.C01*—were markedly upregulated in the resistant plants. Among them, *BnaNPR3.A02* exhibited the most prominent expression divergence between the R and S plants, with sustained upregulation in the resistant line and pronounced suppression in the susceptible line. Based on these results, we heterologously overexpressed *BnaNPR3.A02* in *Arabidopsis thaliana* and generated *npr3* CRISPR/Cas9 knockout mutants (*KO-npr3-1*, *KO-npr3-2*) with base deletion (Supplementary Figure S6) to assess the conserved role of NPR3 in clubroot resistance.

### 2.11. BnaNPR3.A02 Negatively Regulates Arabidopsis Resistance to Clubroot Disease

**Figure 8:**
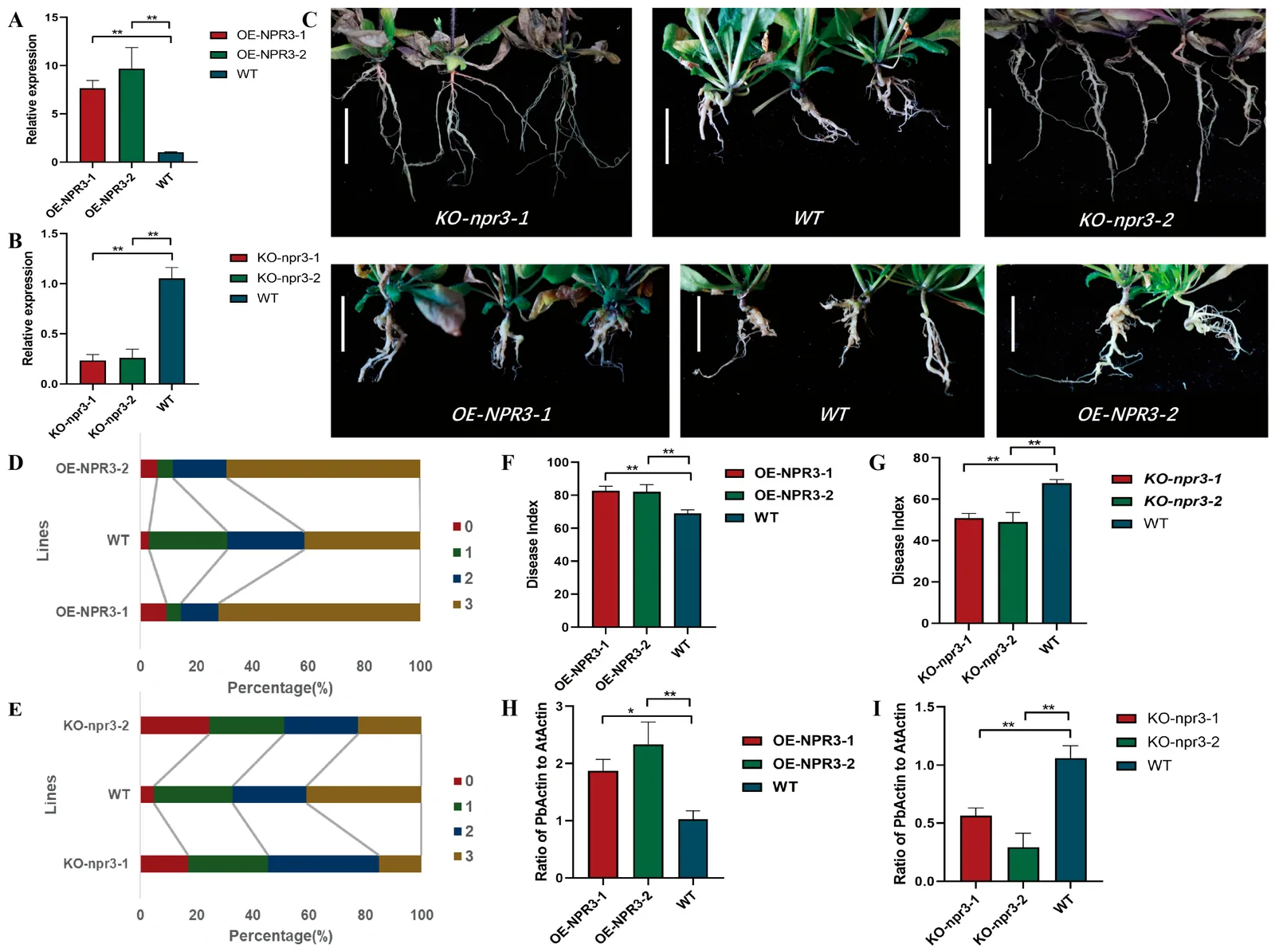
*BnaNPR3.A02* negatively regulates resistance to clubroot disease in Arabidopsis. (**A**) *BnaNPR3.A02* expression in wild-type (WT) and overexpression (OE-NPR3-1, OE-NPR3-2) lines. (**B**) *AtNPR3* expression in WT and *npr3* knockout (KO-npr3-1, KO-npr3-2) lines. (**C**) Disease phenotypes of WT, OE-NPR3, and KO-npr3 plants at 28 days post-inoculation (dpi) with *P. brassicae*. Scale bar: 1 cm. (**D**,**E**) Percentage distribution of plants among disease severity grades for each genotype. Data are presented as means from three independent experiments. Individual biological replicate values are provided in Supplementary Table S4. Percentages are presented for visualization only and were not used as continuous variables for statistical testing. (**F**,**G**) Disease index values of WT, OE-NPR3, and KO-npr3 lines. (**H**) Relative pathogen biomass (*P. brassicae* PbActin/plant Actin7) in WT and OE-NPR3 lines. (**I**) Relative pathogen biomass in WT and KO-npr3 lines. For (**A**,**B**,**F**–**I**), data are presented as means ± SD from three independent experiments. One-way ANOVA followed by Tukey’s HSD post-hoc test was used for multiple comparisons among genotypes. Asterisks indicate significant differences (* *p* < 0.05, ** *p* < 0.01).

qRT-PCR analysis was first performed to determine the expression levels of the target genes in the transgenic and mutant lines. The results revealed that *BnaNPR3.A02* was significantly upregulated in the OE-NPR3 lines compared with the wild type (WT), whereas *AtNPR3* expression in the KO-npr3 lines was significantly reduced relative to WT (Figure 8A,B). We compared the disease severity of transgenic lines overexpressing *BnaNPR3.A02* (OE-NPR3-1, OE-NPR3-2), *npr3* knockout mutants (KO-npr3-1, KO-npr3-2), and wild-type (WT) plants at 28 days post-inoculation with *P. brassicae*. Phenotypic observation revealed that the OE lines exhibited severely damaged root systems, with fibrous roots almost completely absent and pronounced swelling of the main root. In stark contrast, KO mutants showed markedly attenuated symptoms, with only small galls on fibrous roots or occasional minor galls on the main root, while the overall root structure remained relatively intact (Figure 8C). Disease severity grading further demonstrated that the OE-NPR3-1 and OE-NPR3-2 lines had a higher proportion of grade-3 plants, whereas the KO-npr3-1 and KO-npr3-2 lines exhibited a higher proportion of grade-0 plants (Figure 8D,E; Supplementary Table S4). Kruskal–Wallis test followed by Dunn’s post-hoc test confirmed that the OE lines had significantly more severe disease than WT (*p* < 0.01), whereas the KO lines had significantly milder disease than WT (*p* < 0.01), indicating that overexpression of *BnaNPR3.A02* exacerbates disease progression, while its knockout effectively suppresses disease development. The disease index values were consistent with the phenotypic observations: OE-NPR3-1 and OE-NPR3-2 showed indices of 82.73 and 83.03, respectively, significantly higher than that of the corresponding WT control (69.04); conversely, KO-npr3-1 and KO-npr3-2 had indices of 50.83 and 49.58, significantly lower than that of the corresponding WT control (68.63) (Figure 8F,G). Quantification of pathogen biomass further confirmed these results, with OE lines displaying significantly higher pathogen loads than WT, whereas KO mutants exhibited significantly lower loads (Figure 8H,I). These findings demonstrate that overexpression of *BnaNPR3.A02* enhances susceptibility, whereas knockout of *AtNPR3* confers increased resistance, supporting a conserved negative regulatory function of *NPR3* and indicating that *BnaNPR3.A02* acts as a negative regulator in this heterologous system.

### 2.12. Protein–Protein Interaction (PPI) Network and Co-Expression Analysis of BnaNPR3.A02

**Figure 9:**
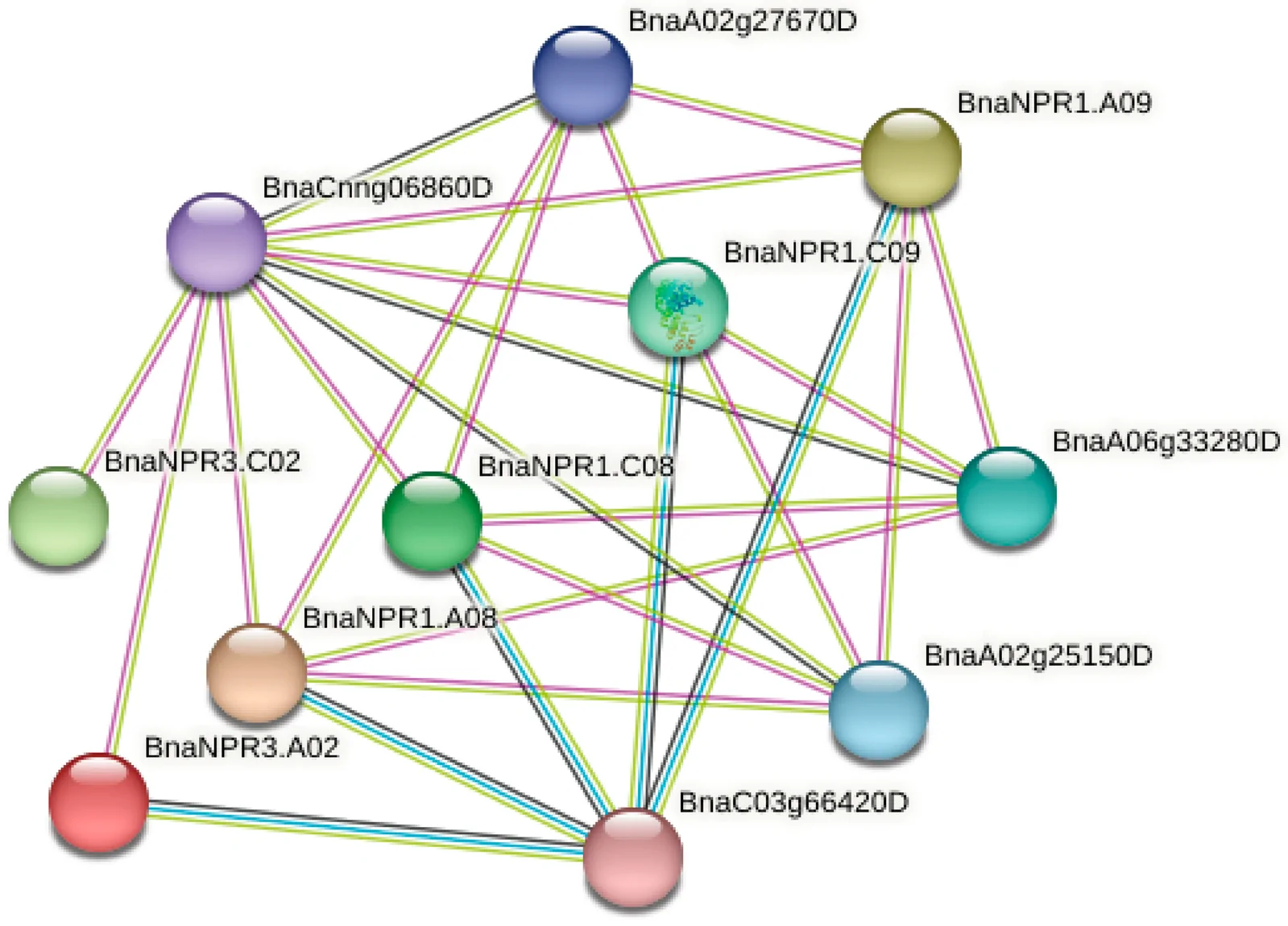
Protein–protein interaction (PPI) network of BnaNPR3.A02. The network was predicted using the STRING database. Nodes represent proteins, and edges indicate predicted functional associations. Different colored edges represent different evidence types in STRING (see https://string-db.org for details).

To better understand the regulatory network of BnaNPR3.A02, we predicted its interacting proteins using the STRING database. Using BnaNPR3.A02 as the query, a network containing 11 interacting protein nodes and 26 edges (all with combined scores ≥ 0.478) was obtained. In this network, the node BnaCnng06860D interacted with multiple proteins (degree = 8) and might serve as a core regulatory node. Notably, BnaNPR3.A02 was predicted to interact with BnaNPR3.C02, suggesting that BnaNPRs can interact with each other (Figure 9). Furthermore, we performed co-expression analysis of the Arabidopsis homolog of *BnaNPR3.A02*, *AtNPR3 (AT5G45110)*, using the ATTED-II database (v13.0). This analysis identified 300 co-expressed genes, which were enriched in GO biological process terms such as response to stress, response to hypoxia, defense response, response to bacterium, and response to chemical stimulus, and in molecular function terms such as protein binding, nucleoside/nucleotide binding, kinase activity, and ion binding (Supplementary Figure S7 and Table S5). These findings indicate that *BnaNPR3.A02* may play important roles in plant defense responses and stress adaptation through interactions with multiple proteins and involvement in extensive co-expression regulatory networks.

## 3. Discussion

NPR3 is a core receptor for the plant defense hormone salicylic acid (SA) and acts as a transcriptional co-repressor in systemic acquired resistance (SAR), thereby functioning as a key regulator of plant immune homeostasis [4,5]. In this study, we performed a genome-wide identification and systematic analysis of the *NPR* family in *Arabidopsis thaliana, Brassica napus* and its diploid progenitors *B. rapa* and *B. oleracea*, identifying 23 *BnaNPR*, 12 *BraNPR*, and 12 *BolNPR* genes. Wang et al. identified 19 *NPR1*-like genes in *B. napus*, whereas our study identified 23 NPR family members; the difference in gene numbers between the two studies is likely attributable to the broader inclusion in our analysis and differences in genome annotation versions. Our study focused on the core NPR family members possessing BTB/POZ, DUF3420, and NPR1-like_C domains, whereas their study included a set of *NPR1*-related sequences with an emphasis on functional validation [23].

Fu et al. reported that the number of NBS-encoding disease resistance genes in *B. napus* (464) was significantly higher than that in its C-genome donor *B. oleracea* (146). The C subgenome of *B. napus* (273 genes) underwent substantial expansion, with only 66.4% of homologous genes retained, whereas the A subgenome (191 genes) maintained a number similar to that of its donor *B. rapa* (202 genes), with a retention rate of 87.1%. These results indicate that *NBS* genes expanded predominantly in the C subgenome following allopolyploidization [29]. Similarly, He et al. identified 287 WRKY transcription factor genes in *B. napus* (139 in the A subgenome and 148 in the C subgenome), with genome duplication, chromosomal/segmental duplications, and tandem duplication collectively driving the expansion of this family, and with duplication events occurring before the merger of the two diploid progenitors [30]. In contrast to these gene families that underwent marked expansion in *B. napus*, the NPR family exhibited a strikingly different evolutionary trajectory. The number of *NPR* genes in *B. napus* (23) was nearly identical to the sum of those in its diploid progenitors—12 in *B. rapa* and 12 in *B. oleracea* (total 24). This near-perfect retention suggests that *NPR* genes have undergone very little loss following allopolyploidization, likely because of their functional importance and dosage balance constraints. The gene balance hypothesis posits that selection acts on gene dosage within regulatory networks and protein complexes to maintain the balanced stoichiometry of interacting proteins; dosage-sensitive genes tend to be retained after polyploidization, whereas dosage-insensitive genes are more likely to be lost [31]. Following allopolyploidization, plant genomes often undergo extensive restructuring, with one subgenome frequently gaining advantages in expression and retention—a phenomenon known as subgenome dominance, which may be driven by factors such as transposon silencing. In *B. napus*, subgenomic asymmetry has been observed, with the A subgenome generally exhibiting higher gene expression levels [32]. However, subgenome dominance is not uniformly manifested across all gene families. This is exemplified by the NPR family in the present study, which did not undergo significant gene loss. The near-complete retention of *NPR* genes suggests that their core functions in SA-mediated immune regulation and their integration into complex regulatory networks impose strong dosage balance constraints. It is precisely this constraint that enables *NPR* genes to withstand the gene loss pressure associated with subgenome dominance and to be stably maintained across different subgenomes.

Phylogenetic analysis classified the *NPR* genes from Arabidopsis and the three Brassica species into six subfamilies (I–VI), consistent with the classification in Arabidopsis [7]. All six subfamilies contained genes from all four species, with no species-specific clades. This indicates that the *NPR* gene family has maintained evolutionary conservation during the divergence of Brassica species and the allopolyploidization of *B. napus*. Notably, the homologous genes in *B. napus* clustered with their diploid progenitors—*B. rapa* (the A-genome donor) and *B. oleracea* (the C-genome donor)—further confirming the hybrid origin and allopolyploid history of *B. napus* and providing a direct basis for tracing the functions of different *B. napus NPR* genes back to their diploid ancestors [33]. Thirty-one segmental duplication gene pairs were detected in *B. napus*, whereas 14 and 13 such pairs were found in *B. rapa* and *B. oleracea*, respectively, suggesting that allopolyploidization may have promoted segmental duplications of *NPR* genes in *B. napus*. The Ka/Ks ratios of all homologous gene pairs (205 pairs) were less than 1, indicating that purifying selection is the main driving force maintaining the functional stability of *NPR* genes.

Physicochemical property analysis revealed that all 53 NPR proteins are unstable (instability index > 40) and exhibit diverse subcellular localizations, including the nucleus, chloroplast, cytoplasm, cytoplasm-nucleus, and plasma membrane. The nuclear localization of NPR proteins is consistent with their canonical role in activating pathogenesis-related (PR) gene expression, as classically demonstrated by Kinkema et al. [34], who showed that nuclear import of NPR1 is essential for SAR induction. However, the additional localization of NPR proteins to chloroplasts and other compartments suggests that they may perform non-canonical functions beyond nuclear transcriptional regulation. For instance, Arabidopsis NPR1 can translocate between the chloroplast and nucleus to participate in salt stress responses, and chloroplast-targeted NPR1 can act as a retrograde signal to enhance plant stress tolerance [25,35]. Notably, transgenic expression of *AtNPR1* in rice has been shown to enhance resistance to fungal and bacterial pathogens while compromising tolerance to salt and drought stresses [24], suggesting that *NPR1* may integrate immune and abiotic stress signaling through distinct subcellular compartments. In the present study, several BnaNPR proteins were predicted to be localized in chloroplasts, raising the possibility that they may similarly exert dual functions in both immunity and abiotic stress responses.

Despite the general instability of NPR proteins, they exhibit highly conserved features at the sequence and structural levels. Conserved motif analysis revealed that each subfamily displays a distinct but conserved motif composition: Motifs 8 and 9 were found exclusively in NPR1–NPR4 subfamily members, Motif 10 was restricted to the NPR1 and NPR2 subfamilies, and losses of Motifs 1, 2, and 6 were confined to the NPR3 subfamily. Motif 7 was present in all members of the NPR4, NPR5, and NPR6 subfamilies. These results indicate that genes within the same subfamily may share similar functions. The BTB/POZ domain and NPR1-like_C domain are present in the vast majority of NPR proteins. The BTB/POZ domain is primarily responsible for protein–protein interactions in NPR proteins and is a key structural basis for the formation of NPR1–NPR3/NPR4 complexes and for the interaction of NPR proteins with TGA transcription factors [36]. In this study, with the exception of five NPR5/6 members that contained only the DUF3420 domain, the remaining 48 NPR proteins all contained the BTB/POZ domain. All members of Subfamilies I–IV possessed the NPR1-like_C domain, and all except BnaNPR3.C02 and BolNPR3.C02 contained ankyrin repeats. These findings indicate that *B. napus* NPR proteins have largely retained molecular interaction capabilities similar to those of Arabidopsis NPR proteins during evolution, while also revealing structural divergence in the NPR5/6 subfamilies that may be related to their distinct functions in development. Combined with the observed structural conservation of core domains across species [19], these findings suggest that NPR-mediated defense responses may be evolutionarily conserved in both monocots and dicots.

Gene structure analysis revealed that the number of exons in *NPR* genes varies considerably, ranging from 1 to 18, with the two- or four-exon structures being the most common, together accounting for 32 genes. Notably, all members of the NPR5 and NPR6 subfamilies contained only one or two exons, suggesting a relatively simple gene structure in these two subfamilies. This diversity in exon number suggests that *NPR* genes may produce different transcript variants through alternative splicing, thereby expanding their functional diversity. Previous studies have shown that alternative splicing of *OsNPR3* in rice produces a truncated protein variant that compromises defense gene activation by sequestering OsNPR1, thereby enhancing susceptibility to pathogens [18]. Therefore, the observed differences in exon numbers among *NPR* genes suggest that similar post-transcriptional regulatory mechanisms may exist in *B. napus NPR* genes, a possibility that awaits further investigation.

Promoter cis-element analysis revealed that the promoters of most *BnaNPR* genes are enriched with multiple hormone-responsive (SA, JA, ABA, GA, IAA) and stress-responsive (drought, low temperature, hypoxia, wounding) elements. This finding is consistent with the differential expression patterns of BnaNPRs under phytohormone and abiotic stress treatments, suggesting that BnaNPRs may serve as integrative hubs within hormone signaling networks and play important roles in multiple stress responses.

Under hormone stress conditions, jasmonic acid (JA) exerted a widespread inhibitory effect in leaves, whereas both abscisic acid (ABA) and JA exhibited similar suppressive effects in roots. This suppression is particularly significant, as JA signaling has been reported to directly repress *NPR1* gene transcription. The underlying mechanism involves the classical antagonistic relationship between the SA and JA signaling pathways. As a core regulator of the SA signaling pathway, NPR1 is essential for SA-mediated suppression of JA signaling. In the presence of SA, NPR1 physically interacts with MYC2, a key transcription factor in the JA pathway, thereby blocking its activation of downstream JA-responsive genes [37]. Consequently, JA application activates the JA signaling pathway, which in turn attenuates the antagonistic SA signaling pathway, leading to repression of *NPR1* expression and its mediated defense responses. This dynamic regulatory mechanism reflects the flexibility of plants in conducting “cross-talk” among different hormone signals to optimize defense strategies.

Under abiotic stress conditions, drought triggered the most extensive transcriptional responses in leaves, which may be associated with SA-mediated NPR1-dependent proline synthesis contributing to redox regulation under drought stress [38]. Salt stress induced upregulation of multiple BnaNPR members, consistent with reports that *AtNPR1* is induced under drought and salt stress in Arabidopsis, as overexpression of *AtNPR1* suppresses salt- and drought-responsive gene expression in rice [24]. Freezing stress (–4 °C) generally suppressed the expression of most *BnaNPR* genes, whereas cold stress (4 °C) selectively induced the upregulation of multiple genes including *BnaNPR1.A08* and *BnaNPR1.C08*, which may be attributed to NPR1-mediated cold acclimation through interaction with ICE1 and TGA3, as well as through an HSFA1-mediated pathway that operates independently of SA and TGA factors [39]. *BnaNPR3.C07* reached its peak expression in roots under drought and osmotic stress, far exceeding that of other BnaNPR members, suggesting a specialized role in root water stress responses. Collectively, these findings demonstrate that *BnaNPR* genes integrate multiple hormone and environmental signals through complex regulatory mechanisms, positioning this family as a key hub coordinating growth, stress adaptation, and immune homeostasis.

GO annotation revealed that *BnaNPR* genes can be divided into two major functional groups: NPR1-4 members are enriched in JA/SA-mediated defense signaling pathways and immune regulation, whereas NPR5-6 members are primarily involved in floral organ development and morphogenesis. Among all *BnaNPR* genes examined, *BnaNPR3.A02* exhibited the most striking expression divergence between the resistant (R) and susceptible (S) plants upon *P. brassicae* infection—showing sustained upregulation in the R plants and consistent repression in the S plants. It should be noted that the R and S plants were selected from a BC_3_ segregating population rather than from near-isogenic lines, and genetic segregation at other loci may also contribute to the observed expression differences. Given that NPR3 functions as a negative regulator of SA signaling, its upregulation in the R plants may reflect a feedback attenuation mechanism to prevent overactivation of immune responses, whereas its sustained suppression in the S plants suggests a defective SA signaling node that fails to induce this negative regulator. The coexistence of sustained upregulation of *BnaNPR2.A01* and *BnaNPR2.C01* with persistent suppression of *BnaNPR3.A02* in the S plants further supports the notion of a dysregulated defense network in the susceptible genotype. The differential expression of *BnaNPR3.A02* between the R and S plants may arise from promoter polymorphisms affecting transcription factor binding, genotype-specific DNA methylation modulating chromatin accessibility, or differential recognition of cis-elements by pathogen-responsive transcription factors. The underlying regulatory circuits remain to be elucidated through future promoter comparison, ATAC-seq, and methylation profiling.

Transgenic functional validation in a heterologous Arabidopsis system supported the negative regulatory role of *NPR3*. Overexpression of *BnaNPR3.A02* in Arabidopsis led to increased disease severity, a higher disease index, and elevated pathogen biomass, whereas the *npr3* knockout mutant exhibited significantly enhanced resistance. These findings are consistent with previous reports that *AtNPR3* functions as an SA receptor and negatively regulates immunity [11,12,13]. This functional conservation echoes the finding in wheat that targeted degradation of *TaNPR3* enhances disease resistance [17], suggesting that *NPR3*-mediated negative regulation of immunity may be a general mechanism in crops.

Protein–protein interaction network prediction showed that BnaNPR3.A02 may interact with BnaNPR3.C02. Although STRING database predictions provide initial interaction clues, these need to be further validated by yeast two-hybrid or co-immunoprecipitation (Co-IP) experiments. Co-expression analysis of the Arabidopsis homolog *AtNPR3* (*AT5G45110*) identified 300 co-expressed genes significantly enriched in defense- and bacterium-related GO terms, further supporting its role in immune regulation. This pattern is consistent with the established SA signaling framework in which NPR1 positively regulates defense genes whereas NPR3/4 attenuate immunity via promoting NPR1 degradation [9,11,12,13].

Based on the known function of NPR3 as a transcriptional co-repressor in SA signaling, we propose a working model: under normal conditions, BnaNPR3.A02 functions as a negative regulator of SA signaling, moderately suppressing defense activation to maintain immune homeostasis. In the resistant plants, upon *P. brassicae* infection, *BnaNPR3.A02* is transiently upregulated as part of a feedback attenuation mechanism, preventing overactivation of immune responses that could cause detrimental effects on the host. In the susceptible plants, however, *BnaNPR3.A02* remains consistently suppressed throughout the infection course, reflecting a defective SA signaling node that fails to induce this negative regulator, resulting in dysregulated defense signaling and subsequent disease progression.

In summary, this study provides a genome-wide identification and comparative analysis of the *NPR* gene family across *B. napus*, its diploid progenitors *B. rapa* and *B. oleracea*, and the model plant *A. thaliana*, complementing and extending the previous work by Wang et al. [23]. We reveal the evolutionary characteristics and expression divergence of this family after allopolyploidization and provide evidence for a conserved negative regulatory role of *NPR3* in a heterologous Arabidopsis system. These findings identify *BnaNPR3.A02* as a candidate for future functional validation and potential gene editing in *B. napus* breeding programs.

## 4. Materials and Methods

### 4.1. Identification of NPR Family Genes in Three Brassica Species

The amino acid sequences of the AtNPR family were obtained from the TAIR database (https://www.arabidopsis.org/, accessed on 22 August 2026) [40]. The amino acid sequences for *B. napus* (Dar_V5/), *B. rapa* (Chiifu_V3.0/), and *B. oleracea* (HDEM_V1.0/) were retrieved from the Brassicaceae database (http://brassicadb.cn/#/Download/, accessed on 22 August 2026) [41]. AtNPR family amino acid sequences were used as seed sequences to perform BLASTP search in the three Brassica species [42]. Subsequently, the HMMER search program (v3.3) was used to identify non-redundant hits with an E-value threshold of <1 × 10^−5^, based on the Hidden Markov Model (HMM) profile (PF00651) retrieved from the Pfam database (https://www.ebi.ac.uk/interpro/entry/pfam/, accessed on 22 August 2026) [43]. Hits from BLASTP and HMMER were merged, and redundant sequences were removed. To further validate the NPR family members, we performed a search using the NCBI Conserved Domain Search (CDD) (https://www.ncbi.nlm.nih.gov/Structure/cdd/wrpsb.cgi, accessed on 22 August 2026) to confirm the presence of the Broad-Complex, Tramtrack and Bric a brac (BTB) and Pox virus and Zinc finger (POZ) domain or the DUF3420 superfamily [44].

### 4.2. Physicochemical Properties and Subcellular Localization of NPR Proteins

The physicochemical properties of NPR proteins from *A. thaliana*, *B. napus*, *B. rapa* and *B. oleracea* were analyzed using the ProtParam tool on the ExPASy server (https://web.expasy.org/protparam/, accessed on 22 August 2026) [45]. The subcellular localization of NPR proteins in the three Brassica species was predicted using WoLF PSORT (https://wolfpsort.hgc.jp/, accessed on 22 August 2026), with the highest-scoring prediction being selected [46].

### 4.3. Phylogenetic Analysis of the NPR Family in Three Brassica Species

The NPR family protein sequences were subjected to multiple sequence alignment using the MAFFT online server (v7.520, https://mafft.cbrc.jp/alignment/server/, accessed on 22 August 2026) [47]. Maximum likelihood (ML) phylogenetic trees were constructed using IQ-TREE software (v3.0.1) [48]. The best-fit model was automatically determined using the ModelFinder function implemented in IQ-TREE (with the -m MFP parameter). Branch support was assessed using 1000 ultrafast bootstrap (UFBoot) replicates.

### 4.4. Analysis of Conserved Motifs, Domains and Gene Structure of NPRs in Three Brassica Species

Conserved motifs in NPR proteins were predicted using the MEME suite (http://meme-suite.org/) [49], with the number of motifs set to 12 and the motif length ranging from 6 to 50 amino acids. Conserved domains were annotated using the InterPro database (https://www.ebi.ac.uk/interpro/, accessed on 22 August 2026) and NCBI Batch CDD-search (https://www.ncbi.nlm.nih.gov, accessed on 22 August 2026) [43,44]. In addition, gene structure analysis was conducted based on gene annotation information. Gene structures, conserved motif distributions, and domain architectures were visualized using ggtree v3.14.0 in RStudio [50].

### 4.5. Gene Duplication and Ka/Ks Analyses of NPR Genes in Three Brassica Species

The chromosomal localization of *NPR* genes in the three Brassica species was extracted from the GFF files and visualized using TBtools-II [51]. Intra-species and inter-species synteny analyses for *B. napus*, *B. rapa,* and *B. oleracea* were performed using MCScanX to identify segmental duplications and tandem duplications [52]. The non-synonymous (Ka) and synonymous (Ks) substitution ratios between duplicated gene pairs were calculated using TBtools [51].

### 4.6. Cis-Regulatory Element Analysis in the Promoter Region of NPR Genes in Three Brassica Species

The 2000 bp upstream sequences from the ATG start codon were extracted and submitted to the PlantCARE database (https://bioinformatics.psb.ugent.be/webtools/plantcare/html/, accessed on 22 August 2026) for cis-regulatory element identification [53]. The distribution of promoter elements was visualized using heatmaps generated in TBtools-II [51].

### 4.7. Expression Profiling of BnaNPRs Across Tissues and in Response to Abiotic Stresses and Phytohormones in B. napus

The spatiotemporal and stress-responsive expression patterns of *BnaNPRs* in the *B. napus* cultivar ZS11 were analyzed using transcriptomic data from the BnIR database (https://yanglab.hzau.edu.cn/BnIR, accessed on 22 August 2026) [54]. For abiotic stress treatments, seedlings were exposed to salt (200 mM NaCl), drought (air-drying), temperature extremes (−4 °C freezing, 4 °C cold, 38 °C heat), or osmotic stress (300 mM mannitol). Root and leaf samples were collected at 0.25, 0.5, 1, 3, 6, 12, and 24 h post-treatment. For phytohormone responses, plants were treated with 10 µM auxin (IAA), 1-aminocyclopropane-1-carboxylic acid (ACC), gibberellin (GA), abscisic acid (ABA), trans-zeatin (TZ), jasmonate methyl ester (JA-Me), and brassinolide (BL), with sampling at 0.5, 1, 3, and 6 h after application. Data normalization and heatmap visualization were performed using TBtools [51].

### 4.8. RNA Extraction and Quantitative Real-Time PCR (qRT-PCR)

Total RNA was extracted from *B. napus* line “1603” and *A. thaliana* root tissues using the TIANGEN RNAprep Pure Plant Kit (DP432) according to the manufacturer’s instructions. Genomic DNA was removed and first-strand cDNA was synthesized using the TransGen EasyScript One-Step gDNA Removal and cDNA Synthesis SuperMix. qRT-PCR was performed on a Life Technologies QuantStudio 7 system using the Taq Pro Universal SYBR qPCR Master Mix (Vazyme, Q712). *BnaActin* and *AtActin7* were used as reference genes, respectively. The stability of these reference genes under our experimental conditions has been validated in our previous study [55]. Relative expression levels were calculated using the 2^−ΔΔCt^ method [56]. For each genotype and time point, three independent biological replicates were collected. Each biological replicate was analyzed with three technical replicates. The primer list is provided in Supplementary Table S6.

### 4.9. Construction of Expression Vector and Genetic Transformation

cDNA from *B. napus* line “1603” was used as a template to clone the *BnaNPR3.A02* gene. The overexpression vector pCAMBIA1300-eGFP was digested with BglII and XbaI. The target fragment was then ligated into the digested vector using the ClonExpress II One Step Cloning Kit (Vazyme, C112-01). The primers are listed in Supplementary Table S6. For CRISPR/Cas9-mediated gene editing, single-guide RNA (sgRNA) targeting *AtNPR3* was designed using CRISPR-P v2.0 (http://crispr.hzau.edu.cn/CRISPR2/, accessed on 20 July 2024) [57]. Complementary oligonucleotides were synthesized to construct the knockout vector. The target fragment was amplified from the pcbc-c1 vector using specific primers (Supplementary Table S6) and inserted into the BsaI-digested PBSE vector via homologous recombination.

After verifying the positive clones by sequencing and visualizing the base deletion patterns of the mutants using the IBS online tool (https://ibs.cebitec.uni-bielefeld.de/, accessed on 22 August 2026), the recombinant plasmid was transformed into Agrobacterium tumefaciens strain GV3101, which was subsequently introduced into *A. thaliana* plants using the floral dip method [58,59]. Transgenic plants were selected on 1/2 Murashige and Skoog (MS) medium containing 25 µg/mL hygromycin until homozygous lines were obtained.

### 4.10. Plant Growth Conditions and P. brassicae Inoculation

Seeds of *Arabidopsis thaliana* and *B. napus* line “1603” were obtained from the rapeseed breeding research group of Northwest A&F University. The *B. napus* line “1603” is a BC_3_ segregating population derived from an artificial *B. napus* hybrid between a clubroot-resistant cabbage (*B. oleracea*) and a susceptible Chinese cabbage (*B. rapa*), followed by three backcrosses to an elite field parent; this population is not a near-isogenic line and may still segregate at loci other than the target clubroot resistance locus. Seeds were sown separately in pots containing a substrate mixture of soil:vermiculite:perlite = 3:1:1, and the plants were grown in a growth chamber at 24 °C under a photoperiod of 16 h light (20 °C)/8 h dark. At the 4–5–leaf stage, a large number of seedlings were inoculated with the *P. brassicae* suspension prepared according to the method of Williams [60], and each plant was labeled individually. Non-destructive sampling was performed at 0, 7, 14, 21, and 28 days post-inoculation (dpi): at each time point, a small portion of root tissue was excised from each plant, and the same plants were returned to the growth chamber and maintained until 28 dpi for phenotype grading. At 28 dpi, plants with Grade 0 were classified as resistant (R) and plants with Grade 2–3 as susceptible (S). For each time point, three individual R plants and three individual S plants were selected for qRT-PCR analysis, with each plant treated as one biological replicate. Root tissues of *A. thaliana* were collected at 0 dpi for qRT-PCR analysis of *BnaNPR3.A02* and *AtNPR3* expression. All samples were immediately frozen in liquid nitrogen and stored at −80 °C.

### 4.11. Phenotypic Identification and P. brassicae Biomass Quantification

The roots of wild-type plants (WT), overexpression (OE) lines (OE-NPR3-1, OE-NPR3-2), and CRISPR/Cas9 mutants (KO-npr3-1, KO-npr3-2) were carefully pulled out and washed to observe for tumor size and distribution on the roots at 28 dpi. The severity of clubroot disease was classified into four grades:

Grade 0—no disease symptoms;

Grade 1—no tumor on the main root, with few and small galls on lateral roots;

Grade 2—slight infection on the main root or severe galls on lateral roots;

Grade 3—large galls on the main root, with almost no lateral root formation.

The percentage of each severity grade for a given genotype was calculated as:

(Number of plants in that grade/Total number of plants in that replicate) × 100%.

Data are presented as means from three independent experiments. In addition, the disease index (DI) was calculated according to the formula: DI (%) = [Σ (Grade_i_ × n_i_)/(N × Highest Grade)] × 100% [61]. All phenotypic assessments were performed in three independent experiments, with each experiment comprising at least 30 individual plants per genotype.

For quantification of *P. brassicae* biomass, the roots of inoculated plants were harvested at 28 dpi. Total DNA was extracted using the Hi-DNAsecure Plant Kit (TIANGEN) according to the manufacturer’s instructions. The biomass of *P. brassicae* was determined by quantitative PCR (qPCR) using primers specific to the *P. brassicae* actin gene (*PbActin*) with the SYBR Premix Ex Taq Kit (Takara). *Arabidopsis thaliana* actin (*AtActin7*) was used as the internal reference gene for normalization, as appropriate [62]. The primers used for biomass measurement are listed in Supplementary Table S6. All measurements were performed with three biological replicates.

### 4.12. GO Annotation of BnaNPRs and Identification of BnaNPR3.A02 Co-Expressed Genes

GO annotation of the *BnaNPRs* was performed using the BnIR database GO enrichment program (https://yanglab.hzau.edu.cn/BnIR/GO, accessed on 22 August 2026) [54]. To identify co-expressed genes of the *A. thaliana* homolog *AtNPR3*, the ATTED-II v13 database (https://atted.jp/, accessed on 22 August 2026) was used [63]. GO enrichment analysis of the co-expressed genes was conducted in the TAIR database (https://v2.arabidopsis.org/tools/go_term_enrichment.jsp, accessed on 22 August 2026) [40].

### 4.13. Statistical Analysis

Statistical analyses and data visualization were performed using R software (v4.3.1) (https://www.r-project.org/, accessed on 22 August 2026) and GraphPad Prism 8. All data are presented as means ± standard deviation (SD) from three biological replicates. For multiple comparisons among three or more groups, one-way analysis of variance (ANOVA) followed by Tukey’s HSD post-hoc test was applied. Significant differences between groups are indicated with asterisks (* *p* < 0.05, ** *p* < 0.01). For disease severity grade distributions, individual plants were assigned ordinal grades (0–3). The original ordinal grade values of all individual plants were pooled within each genotype and analyzed using the Kruskal–Wallis test followed by Dunn’s post-hoc test with Bonferroni correction. Percentages were calculated for each biological replicate and are presented for visualization only; they were not used as continuous variables for statistical testing.

## 5. Conclusions

In this study, we identified 23, 12, and 12 NPR family members in *B. napus*, *B. rapa*, and *B. oleracea*, respectively, and classified them into six subfamilies (I–VI). Phylogenetic tree, conserved domain, and gene structure analyses revealed the conservation and diversity of this gene family. Spatiotemporal expression analysis demonstrated that *BnaNPRs* exhibit tissue-specific expression patterns. Promoter analysis, along with expression profiling under phytohormone and abiotic stress treatments, indicated that BnaNPRs are involved in various stress responses. Furthermore, expression analysis under *Plasmodiophora brassicae* stress highlighted the potential role of *BnaNPR3.A02* in clubroot resistance. Overexpression of this gene in Arabidopsis significantly enhanced susceptibility compared with WT, whereas knockout of *AtNPR3* increased resistance, supporting that *BnaNPR3.A02* functions as a conserved negative regulator in a heterologous system. Protein–protein interaction (PPI) network and co-expression analyses further suggested that *BnaNPR3.A02* may participate in plant immune responses through interaction with other NPR members and involvement in defense-related biological processes.

## Data Availability

The RNA-seq data from *B. napus* ZS11 under various hormone and stress treatments are available through the BnIR database (https://yanglab.hzau.edu.cn/BnIR, accessed on 22 August 2026) developed by the Yang Lab at Huazhong Agricultural University.

## Supplementary Materials

The following supporting information can be downloaded at: https://www.mdpi.com/article/10.3390/plants15203149/s1, Figure S1: Detailed distribution of conserved motifs in NPR proteins. The 12 conserved motifs identified by MEME are shown as sequence logos. The height of each letter represents the degree of conservation at that position, with taller letters indicating higher conservation; Figure S2: Tissue expression patterns of *BnaNPR* genes in *Brassica napus* (ZS11). Heatmap showing the TPM (Transcripts Per Million) values of 21 *BnaNPR* genes across various tissues. Color scale: blue (low expression) to red (high expression); Figure S3: Spatiotemporal expression of BnaNPRs during leaf, seed, and silique development. Expression dynamics during leaf development (leaf 1 to leaf 23), seed development (14–64 days after flowering, DAF), and silique development (2–60 DAF). Color scale: blue (low expression) to red (high expression); Figure S4: Expression responses of BnaNPRs to phytohormone treatments in leaves (A) and roots (B). Heatmaps display the TPM (Transcripts Per Million) values of BnaNPRs. Hormone treatments: IAA, ACC, GA, ABA, TZ, JA-Me, BL (each 10 µM). Sampling times: 0.5, 1, 3, 6 h. Color scale: blue (low expression) to red (high expression); Figure S5: Expression profiles of BnaNPRs under abiotic stresses. Heatmaps showing the TPM (Transcripts Per Million) values of BnaNPRs in leaves (A) and roots (B) under salt (200 mM NaCl), drought, freezing (–4 °C), cold (4 °C), heat (38 °C), and osmotic (300 mM mannitol) stresses. Sampling times: 0.25, 0.5, 1, 3, 6, 12, 24 h. Color scale: blue (low expression) to red (high expression); Figure S6: Alignment of genomic DNA sequences of npr3 knockout lines (KO-*npr3*-1 and KO-*npr3*-2) with the reference sequence of *AtNPR3*; Figure S7: Co-expression analysis of *AtNPR3* (the Arabidopsis homolog of *BnaNPR3.A02*). (A) GO enrichment analysis of co-expressed genes in the biological process (BP) category. (B) GO enrichment analysis of co-expressed genes in the molecular function (MF) category. *p*-values are indicated by the color scale, and the number of genes is shown by dot size; Table S1: Physicochemical parameters and subcellular localization of *NPR* genes in three Brassica species; Table S2: Ka/Ks values of *NPR* gene pairs in three Brassica species; Table S3: GO Enrichment Analysis of BnaNPRs; Table S4: Disease severity grades in OE and KO lines and their corresponding WT controls; Table S5: GO Enrichment Analysis of *AtNPR3* co-expression genes; Table S6: Primers used for analysis.
